# Characterization of long-chain acyl-CoA synthetases which stimulate secretion of fatty acids in green algae *Chlamydomonas reinhardtii*

**DOI:** 10.1186/s13068-016-0598-7

**Published:** 2016-08-31

**Authors:** Bin Jia, Yanzi Song, Min Wu, Baicheng Lin, Kang Xiao, Zhangli Hu, Ying Huang

**Affiliations:** 1 Guangdong Engineering Research Centre for Marine Algal Biotechnology, Guangdong Key Laboratory of Plant Epigenetics, Shenzhen Key Laboratory of Marine Bioresouce and Eco-Enviromental Science, Shenzhen University, Shenzhen, 518060 People’s Republic of China; 2 Key Laboratory of Microbial Engineering at the Institute of Biology, Industrial Enzyme Engineering Technology Research Center, Henan Academy of Sciences, Zhengzhou, 450008 People’s Republic of China

**Keywords:** Long-chain acyl-CoA synthetase, Fatty acids secretion, Antisense knockdown, *Chlamydomonas reinhardtii*

## Abstract

**Background:**

Microalgae biofuel has become the most promising renewable energy over the past few years. But limitations still exist because of its high cost. Although, efforts have been made in enhancement of lipid productivity, the major cost problem in harvesting and oil extraction is still intractable. Thus, the idea of fatty acids (FAs) secretion which can massively facilitate algae harvesting and oil extraction was investigated here.

**Results:**

The cDNAs of two long-chain acyl-CoA synthetases (LACSs) genes were cloned from *Chlamydomonas reinhardtii* and named as *cracs1* and *cracs2*. They showed different substrate adaptation in the yeast complementation experiments. *Cracs2* could utilize FAs C12:0, C14:0, C16:0, C18:0, C16:1 and C18:1, while *crac1* could only utilize substrate C14:0, C16:1 and C18:1. Knockdown of *cracs1* and *cracs2* in *C. reinhardtii* resulted in accumulation of intracellular lipids. The total intracellular lipids contents of transgenic algae q-15 (knockdown of *cracs1*) and p-13 (knockdown of *cracs2*) were 45 and 55 %, respectively higher than that of cc849. Furthermore, FAs secretion was discovered in both transgenic algae. Secreted FAs can reach 8.19 and 9.66 mg/10^9^ cells in q-15 and p-13, respectively.

**Conclusion:**

These results demonstrated the possibility of FAs secretion by microalgae and may give a new strategy of low-cost oil extraction. According to our findings, we proposed that FAs secretion may also be achieved in other species besides *Chlamydomonas reinhardtii* by knocking-down *cracs* genes, which may promote the future industrial application of microalgae biofuels.

**Electronic supplementary material:**

The online version of this article (doi:10.1186/s13068-016-0598-7) contains supplementary material, which is available to authorized users.

## Background

Biofuel has been widely studied over the past few years and is considered to be the most promising renewable energy to ease global energy crisis. However, due to the limited amount of raw materials, the production of traditional biofuel from crops was dramatically constrained. Thus, the new raw materials with low-cost and rich source are in an urgent demand. Microalgae can photoautotrophically grow and produce bulk chemicals, such as lipid. So it is regarded as a powerful raw material and called the 3rd generation biofuels [1, 2]. Though microalgae have a fast growth rate, a high photosynthetic efficiency, and a reduced impact on the environment, limitations still exist that microalgal biofuel is still much more expensive than fossil fuels because of its high producing cost [3]. Efforts can be made in the enhancement of lipid productivity or the decrease of processing cost in harvesting and oil extraction which usually account for 70–80 % of the total cost [4]. Plenty of reports have already focused on the increase in microalgae lipid production by genetic modification; alternatively, very few studies concentrate on reducing processing cost [5, 6]. The idea of FAs secretion which can massively facilitate algae harvesting and oil extraction gives a new sight, and may greatly reduce the whole cost in future.

Acyl-CoA synthetases (ACSs) can activate fatty acids (FAs) into CoA thioesters which can then serve as the substrate for many metabolic pathways, such as fatty acids elongation and desaturation, lipid synthesis, and β-oxidation [7]. According to chain length of their substrates, ACSs were roughly divided into three categories: very long-chain ACSs (>C22), long-chain ACSs (LACSs, C12–C20), and medium-chain ACSs (C6–C10) [8]. ACSs contain two highly conserved amino acid motifs. One motif with 10–12 amino acid residues is supposed for ATP binding activity and the other motif with 25-amino acid residues is considered as ACSs signature motif possible for catalytic activity [9]. In general, distinct classes of LACSs always co-exist in cells, which may have different substrate chain-length specificities or subcellular localizations. For example, *Arabidopsis thaliana* contains at least nine LACSs, of which AtLAC6 and AtLAC7 locate in peroxisome responsible for FAs β-oxidation [10].

It has been reported that LACSs involved in FAs secretion due to the ability of FAs transportation. Disruption of LACSs resulting in FAs exportation into the media was first discovered in *Escherichia coli* [11]. A similar phenomenon was also observed in *Saccharomyces cerevisiae* when native LACSs genes, *faa1* and *faa4* which account for the most of LACSs activity, were both removed [12]. Furthermore, FAs secretion can be greatly enhanced by additional expression of FAs synthesis genes in LACSs gene knockout strains [13, 14].

To date, LACSs have merely been identified in three species of eukaryotic algae. Five LACSs genes (PtACSL1–PtACSL5) were cloned from *Phaeodactylum tricornutum* and only two of them had expected biological activities [15]. TpLACSA and NOLACS (LACSs gene from *Thalassiosira pseudonana* and *Nannochloropsis oculata*) were also isolated and characterized [16, 17]. However, little is known about ACS(s) in model algae *Chlamydomonas reinhardtii*. Furthermore, gene disruption or knockdown experiments were not conducted in none of the abovementioned reports. So it is still not clear whether or not LACSs of microalgae are involved in FAs secretion. The connection between LACS and lipid metabolism needs to be clarified. In this study, using the model algae *C. reinhardtii*, we cloned two novel cDNAs encoding LACSs, constructed mutated algae with native LACSs cDNA knockdown and determined their intracellular lipid and extracellular FAs content.

## Results and discussion

### Identification of potential LACSs genes in *C. reinhardtii*

To clone potential LACSs cDNAs in *C. reinhardtii*, genomeblast was adopted using LACSs genes from related species. Finally, two hypothetical genes were found. Using primers in Additional file 1: Table S1 and RT-PCR, the cDNAs were successfully cloned, verified and submitted to NCBI by the name of *cracs1* (KP751927) and *cracs2* (KP751928). *Cracs1* contains 2004 bp, which is in full agreement with a predicted protein coding sequence (XM-001702895.1) except for a G to C change in base pair 669. *Cracs2* contains 2016 bp and is 399 bp longer than a predicted protein coding sequence (XM-001690784.1). Exon prediction showed *cracs2* is composed of 17 exons. Aligning *cracs2* with genomic sequence, we found that the seventh and eleventh exons with a total length of 399 bp just right fell into the gap region of genomic sequence data. The detailed exon distributions of *cracs1* and *crasc2* were shown in Additional file 2: Figure S1.

**Fig. 1:**
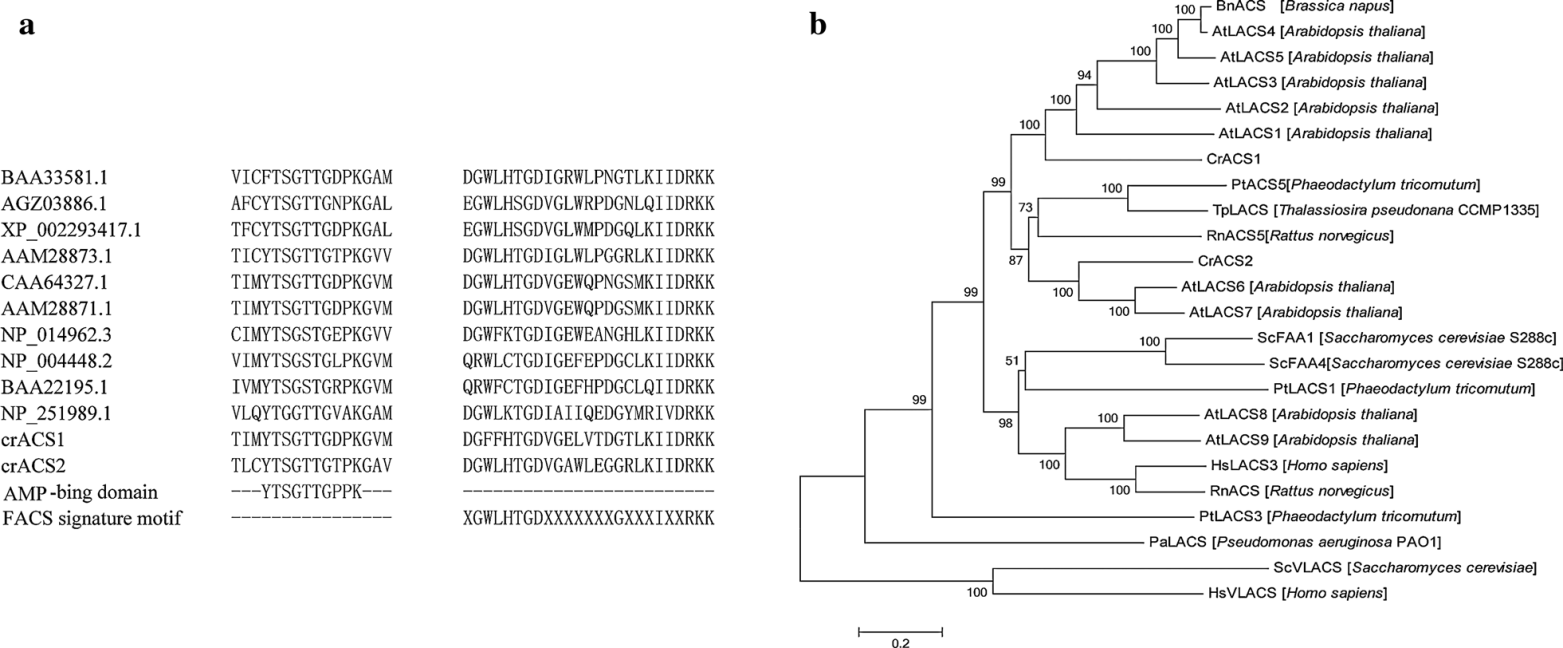
Sequence analysis of deduced amino acids of *C. reinhardtii* LACSs homologs. **a** Multiple sequences alignment of the AMP-Binding domain and consensus sequences of the ACS signature motif. **b** Phylogenetic analysis of CrACSs with LACSs from *A. thaliana*, *Brassica napus, P. tricornutum, T. pseudonana, Rattus norvegicus, S. cerevisiae* and *Homo sapiens*. The tree was constructed using Neighbor-Joining algorithm. The GenBank accession numbers of used sequences are listed in Additional file 6: Table S2. Numbers at branch points are bootstrap percentages derived from 1000 replicates

The deduced encoding proteins of *cracs1* and *cracs2* are 668 and 672 amino acid residues, respectively, with an identity of 40 %. Multiple alignments of their encoding proteins with other known ACSs proteins showed that both *cracs1* and *cracs2* have two highly conserved regions (Fig. 1a). These conserved regions are supposed to be an AMP-binding motif and FACS motif, which are present in all ACSs proteins [8]. This result indicted that *cracs1* and *cracs2* may be the genes encoding ACSs in *C. reinhardtii*.

Phylogenetic tree was constructed and shown in Fig. 1b. Two very-long-chain ACSs, *S. cerevisiae* ScVLACS and *Homo sapiens* HsVLACS, were clustered into an individual branch, while other long-chain ACSs formed a big sub-branch. Therefore, we inferred that CrACS1 and crACS2 should be long-chain ACSs. In addition, CrACS1 and *A. thaliana* AtLACS1–AtLACS5 were grouped in one branch, while, CrACS2 and *A. thaliana* AtLACS6, AtLACS7 were clustered into another branch. Previous studies have already confirmed that AtLACS1 was involved in lipid synthesis and AtLACS6 and AtLACS7 were involved in FAs β-oxidation [10, 18]. As a result, we concluded that functional diversity may exist between CrACS1 and CrACS2 in *C. reinhardtii*.

### In vivo functional analysis of *cracs1* and *cracs2*

To determine whether CrACSs indeed has a biological function, yeast complementation experiments were employed. *Cracs1* and *cracs2* were separately cloned into yeast expression vector pYES2 and then electroporated into *S. cerevisiae* strain YB525. The endogenic LACSs genes *faa1* and *faa4* of YB525 which account for almost 90 % of LACSs activity were disrupted. In the absence of these essential genes, YB525 could not grow on media containing long-chain fatty acids as a sole carbon source when native fatty acid synthesis was inhabited by cerulenin. Under this condition, the growth of YB525 can only be complemented by introducing an active exogenetic LACSs [19]. By adding palmitic acid to dropout uracil medium as the sole carbon source, both CrACSs complemented the growth of YB525, indicating *cracs1* and *cracs2* did encode the active LACSs enzymes.

**Fig. 2:**
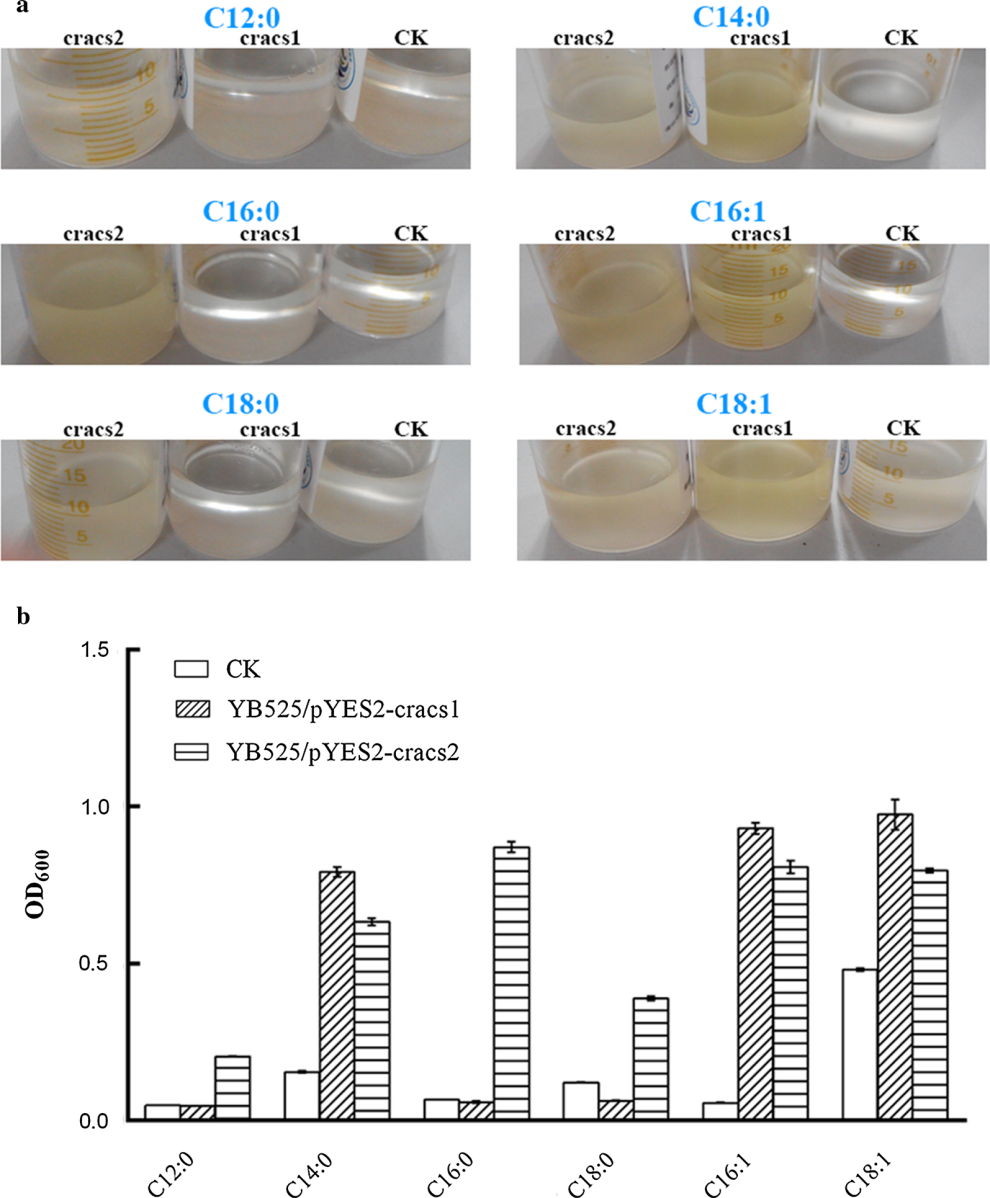
Substrate utilization profiles of yeast strains expressing *cracs1* and *cracs2*. Cells expressing *cracs1* and *cracs2* were named YB525/pYES2-cracs1 and YB525/pYES2-cracs1, respectively. YB525 transformed with blank pYES2 vector was used as control strain. Growth of yeast cells in liquid medium with different FAs as the sole carbon source was observed (**a**) and determined using OD_600_ (**b**) after growing for 240 h

The detailed characteristics of CrACSs was investifgated using six different fatty acids (FAs) as sole carbon source. Four out of six FAs used here were saturated ones (i.e., C12:0, C14:0, C16:0, C18:0) and the other two were unsaturated ones (i.e., hexadecenoic acid, C16:1 and 9-octadecenoic acid, C18:1). It was shown in Fig. 2 clearly that YB525/pYES2-cracs1 grew well in medium containing C14:0, C16:1 or C18:1, but barely grew in medium containing C12:0, C16:0 or C18:0. The growth rates of YB525/pYES2-cracs1 in medium containing C14:0, C16:1 or C18:1 were 5.1, 16.6 and 2 folds higher than that of the control. While, YB525/pYES2-cracs2 grew well in medium containing all selected FAs. The growth rates of YB525/pYES2-cracs2 were 4.2, 4.1, 13.1, 3.2, 14.4 and 1.7 folds higher than that of the control when C12:0, C14:0, C16:0, C18:0, C16:1 or C18:1 was added. Although the control strain showed slight growth in C18:1 maybe resulted from other indigenous LACSs in YB525, such as *faa3* which has a preference for long-chain unsaturated fatty acids [12], the above results still indicated CrACS2 may have a wider FAs adaptability than CrACS1.

**Fig. 3:**
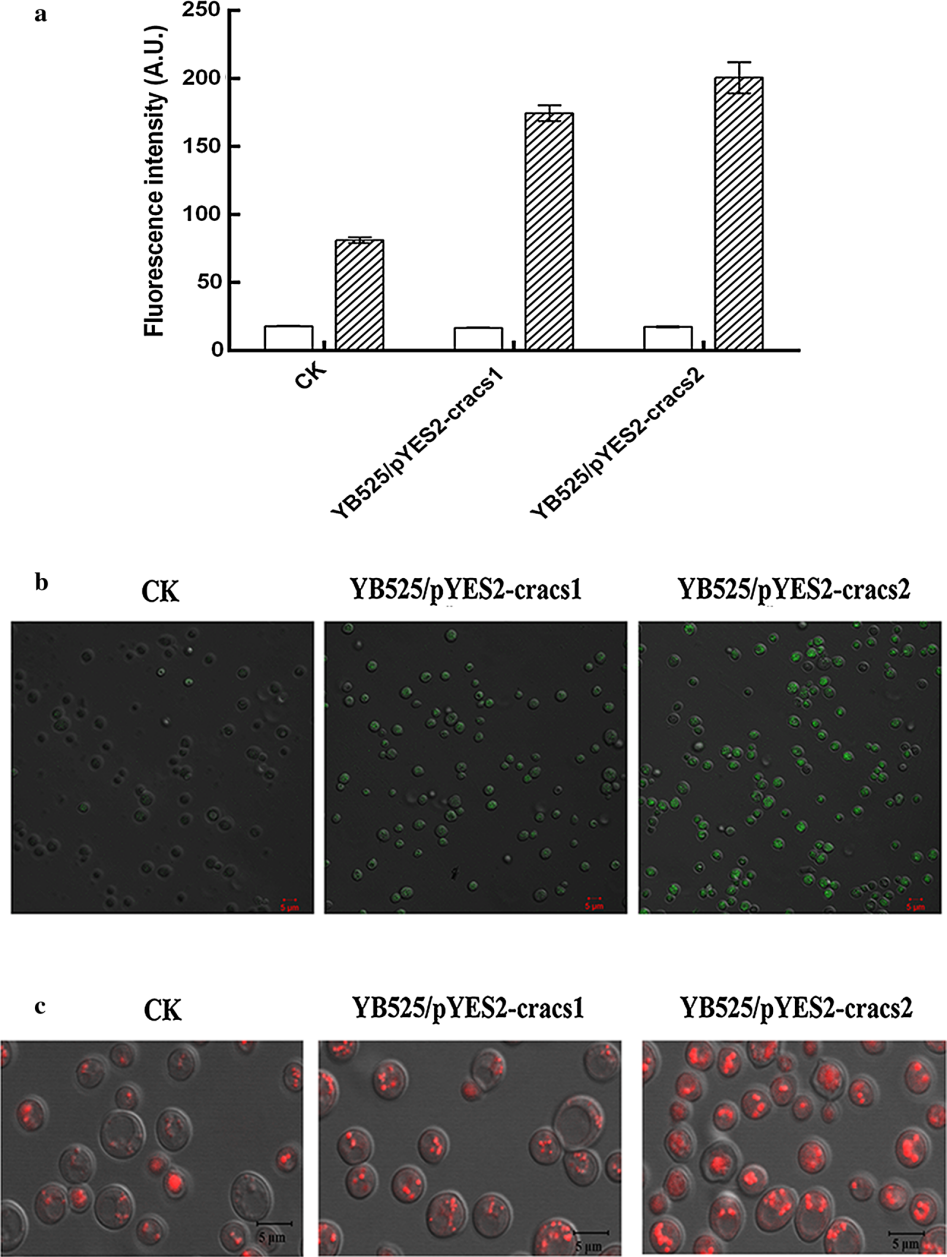
Functional analysis of *cracs* in transgenic yeast YB525. Cells expressing *cracs1* and *cracs2* were named YB525/pYES2-cracs1 and YB525/pYES2-cracs1, respectively. YB525 transformed with blank pYES2 vector was used as control strain. The fatty acid importation ability was determined using C1-BODIPY-C12 as analogues by fluoresce microplate reader (**a**) and confocal microscope (**b**). *White* and *shadow column* indicate the fluorescence intensity before and after C1-BODIPY-C12 was added. **c** Cells stained with *Nile Red* were observed with confocal microscope indicating intracellular lipid accumulation. Each *scale bar* indicates 5 μm

LACSs activities can be determined using fluorescent fatty acid analogues, C1-BODIPY-C12 when de novo FAs synthesis was inhibited in yeast YB525 [20]. Pre-induced YB525, YB525/pYES2-cracs1 and YB525/pYES2-cracs2 were incubated with C1-BODIPY-C12 for 10 min. Then, the fluorescent intensities of the above treated strains were determined, as shown in Fig. 3a. The fluorescent intensities of YB525, YB525/pYES2-cracs1 and YB525/pYES2-cracs2 were 81.2, 174.7 and 200.7, respectively. Therefore, CrACS1 and CrACS2 transformants showed 2.2 and 2.5 folds increase over the control strain. The fluorescent images of the above strains were obtained by the laser confocal microscopy (Fig. 3b). Almost no fluorescent signal of control strain can be detected, while *cracs* transformants showed strong signals intracellular. Besides, YB525/pYES2-cracs2 exhibited a stronger fluorescent signal than that of YB525/pYES2-cracs1. This result implied that CrACS2 may possibly have a higher activity than CrACS1 in yeast.

LACSs have already been reported to be involved in lipid metabolism. So intracellular lipids of YB525 transformants were stained by Nile Red and observed by the laser confocal microscopy. As shown in Fig. 3c, YB525/pYES2-cracs1 and YB525/pYES2-cracs2 exhibited strong fluorescent signals, whereas the control strain showed reduced weak fluorescent signal. Interestingly, a stronger signal was also observed in YB525/pYES2-cracs2 compared with YB525/pYES2-cracs1. This indicated that more lipids were synthesized by CrACS2 involved metabolic pathway and the enzymatic activity of CrACS2 is higher than that of CrACS1.

Nitrogen starvation (NS) is a common method to induce lipid storage in algae. Therefore, we also investigated the relations between NS and *cracs* expression in *C. reinhardtii* by quantitative reverse transcription-PCR (qRT-PCR). Our results showed that the mRNA levels of both *cracs1* and *cracs2* significantly changed in response to NS (Additional file 3: Figure S2). The mRNA level of *cracs1* gradually increased with prolonged NS and increased to 2.2 folds above control after 72 h of NS. Oppositely, the mRNA level of *cracs2* decreased to only 15 % of control after 72 h of NS. From the above results, we concluded that both *cracs1* and *cracs2* encoded active enzymes and may be involved in lipid metabolic pathways in *C. reinhardtii.*

### Antisense knockdown of *cracs* in *C. reinhardtii*

We have already confirmed *cracs1* and *cracs2* are active LACSs genes in *C. reinhardtii.* As a consequence, knockdown experiments of *cracs1* or *cracs2* were conducted to study their roles in lipid metabolism. For this purpose, about 600 bp of *cracs1* or *cracs2* was reversely inserted, respectively, into the downstream of the HSP70-RBCS2 promoter and then transformed into *C. reinhardtii*. The surviving transformed algae cells were selected and screened by PCR to confirm the presence of antisense expression unite (Additional file 4: Figure S3).

**Fig. 4:**
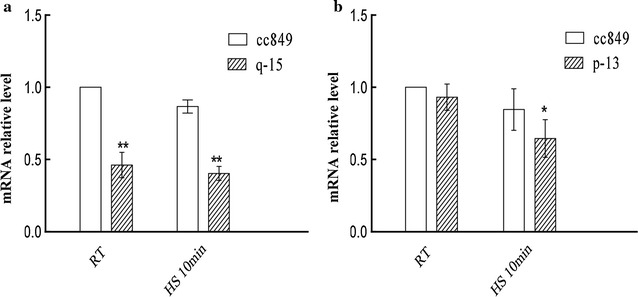
mRNA expression patterns of *cracs1* and *cracs2* in algae q-15, p-13 and control cc849.* RT *room temperature,* HS* heat shock; (*) indicates significant difference (*P* < 0.05) compared to control strain; (**) indicates extremely significant difference (*P* < 0.01) compared to control strain; **a** mRNA expression pattern of *cracs1* in q-15 and cc849; **b** mRNA expression pattern of *cracs2* in p-13 and cc849

Transgenic lines q-15 (antisense knockdown of *cracs1*) and p-13 (antisense knockdown of *cracs2*) were selected and analyzed. Transgenic lines q-15 and p-13 presented similar growth rates compared with wild-type cc849 in the early stage of culture. However, they showed slightly lower growth in the late exponential stage and slightly lower cell density in the stationary stage compared with wild-type (Additional file 5: Figure S4). The transcription abundances of q-15 and p-13 were determined by qPCR. As shown in Fig. 4. After heat induction, the mRNA levels of q-15 and p-13 significantly decreased by 53.78 and 23.67 %, respectively. These results indicated that target *cracs* genes have successfully been knocked-down by antisense RNAs.

### Lipid accumulation in antisense knockdown algae

**Fig. 5:**
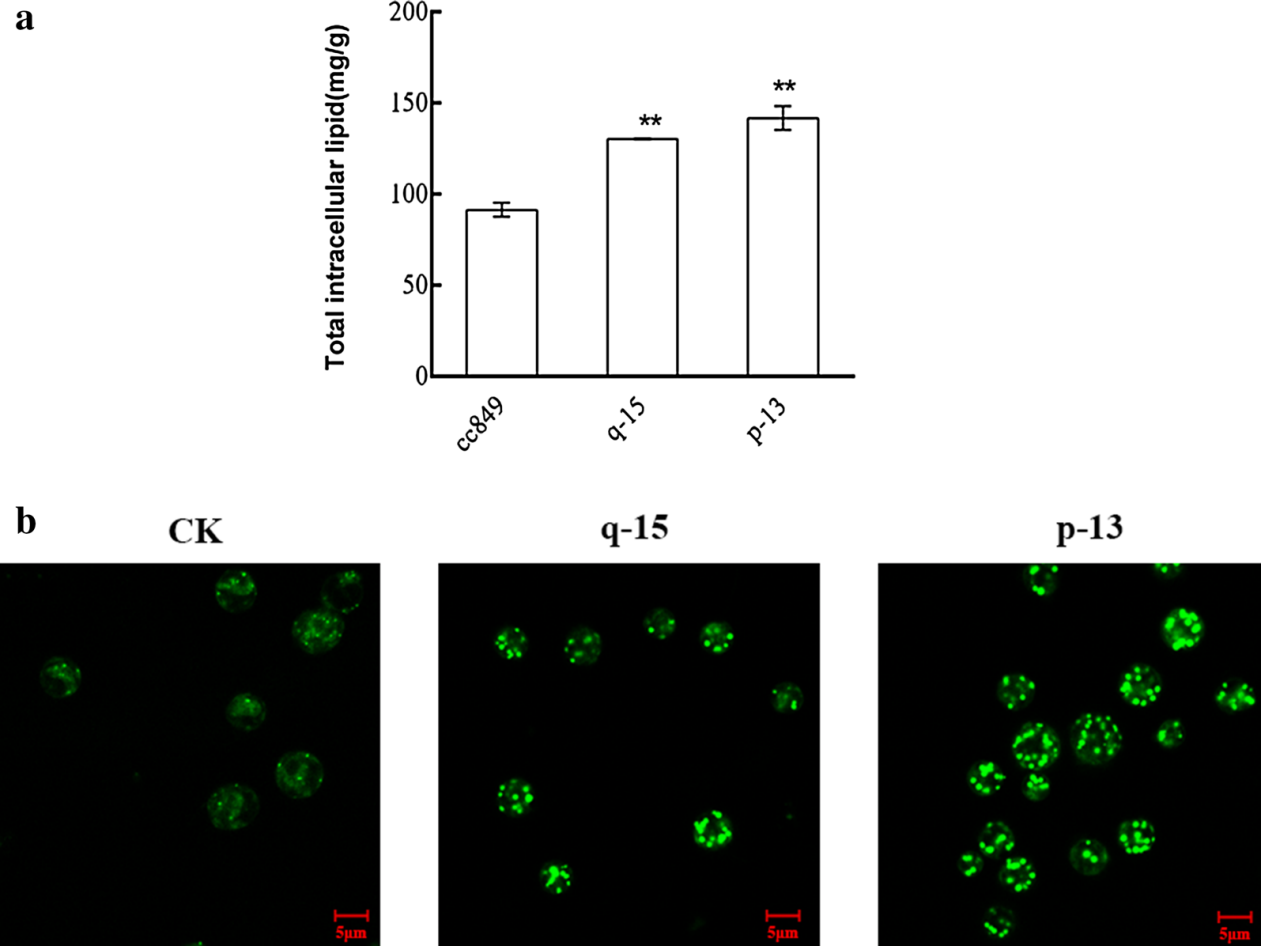
Transgenic algae q-15 and p-13 accumulated more intracellular lipids. (*) indicates significant difference (*P* < 0.05) compared to control strain; (**) indicates extremely significant difference (*P* < 0.01) compared to control strain. **a** the content of total intracellular lipids in q-15, p-13 and control cc849; **b** intracellular lipid droplets stained with BODIPY 505/515 in q-15, p-13 and control were observed by confocal microscope. Each *scale bar* indicates 5 μm

Mid-log stage algae of q-15, p-13 and cc849 were heat induced and re-cultured for another 48 h before lipid determination. The total intracellular lipid contents of q-15 and p-13 were 130.1 and 141.6 mg/g DCW (dry cell weight), respectively, which were 45 and 55 % higher than that of cc489 with a very significant difference (*P* < 0.01, Fig. 5a). Meanwhile, intracellular lipid distribution was also visualized by BODIPY505/515 which can stain neutral lipid. As shown in Fig. 5b, both the amount and size of lipid droplets in transgenic algae were obviously more and bigger than those of wild type cc849. Among them, lipid droplets in p-13 showed the strongest fluorescent signal. The fluorescence intensities of algae were also measured by spectrophotometer. The relative fluorescence intensities of q-15 and p-13 were,29 and 35 %, respectively, higher than that of cc489. This indicated that the increase of neutral lipids may be the main reason for the intracellular lipid content increase. In summary, the above results demonstrated that knockdown of *cracs* caused lipid, mainly TAG, accumulation in *C. reinhardtii*.

**Fig. 6:**
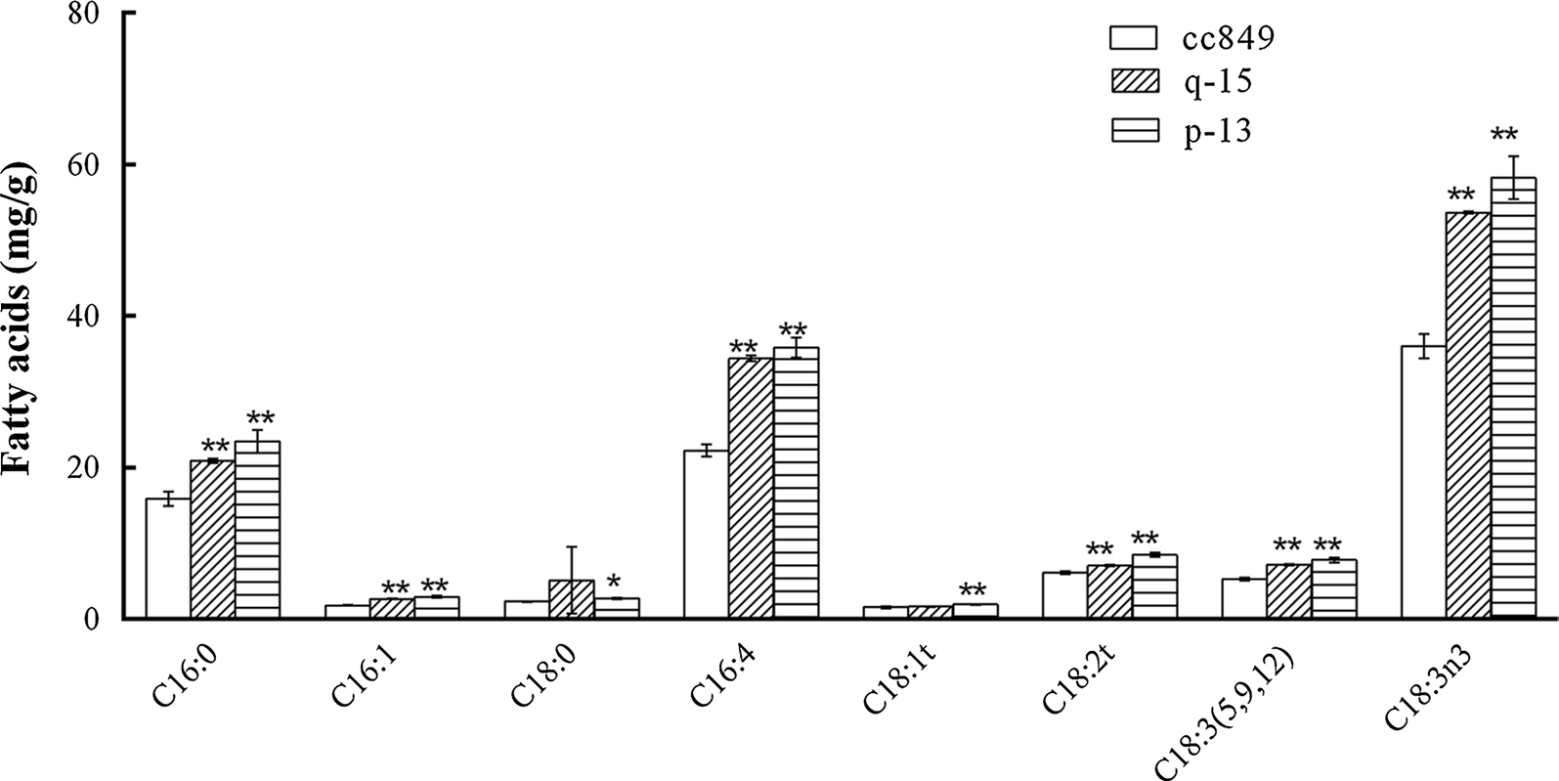
Profile of intracellular fatty acids in algae q-15, p-13, and control cc849 are different. (*) indicates significant difference (*P* < 0.05) compared to control strain; (**) indicates extremely significant difference (*P* < 0.01) compared to control strain

Intracellular lipid profiles of q-15, p-13 and cc849 were analyzed by GC-MASS. As shown in Fig. 6, almost all compositions increased with a significant statistical difference (*P* < 0.01) compared with wild type-cc849. Although major lipids in q-15 and p-13 were still C18:3n3, C16:4 and C16:0, their content showed an obvious increase. Moreover, the percentages of each composition have also varied. The percentages of C18:3n3 and C16:4 in p-13 showed an obvious increase and reached 41.1 and 25.3 %, respectively. It is almost the same case with the percentages of C18:3n3 and C16:4 in q-15. Also, the percentages of C16:0 in q-15 and C18:0 in p-13 exhibited an obvious decrease (*P* < 0.01) compared with that of cc849. These results indicated that knockdown of *cracs* in *C. reinhardtii* may cause unsaturated fatty acid increase. This is opposite to the fatty acid profiles of *acs3* knockout in *Neurospora crassa* and *YAL1* deleted in *Yarrowia lipolytica* [21, 22]. They also showed an obvious increase in saturated FAs C16:0, C18:0 and a decrease in polyunsaturated C18:1, C18:2 and C18:3. However, disruption of *fad* in *E. coli* showed similar results to our results [23]. It has been reported that the substrates activated by LACSs were involved in lipid synthesis on desaturation and elongation or lipid degradation by β-oxidation [24]. It is well-known that the inhibition of β-oxidation can cause the increase of the intracellular lipid content. Therefore, we proposed that knockdown of *cracs* may block β-oxidation, which resulted in the increase in intracellular lipid content. Somehow, it is still not excluded, the possibility of the presence of other LACs genes involved in fatty acids desaturation and elongation.

### FAs secretion in antisense knockdown algae

LACSs disruption induced FAs secretion was already confirmed in *S. cerevisiae* and *E. coli* [25–27], so we investigated whether or not antisense knockdown of CrACSs could induce FAs secretion. We determined the total lipid content in culture medium (TLCCM) by GC-MASS. Interestingly, both transgenic algae q-15 and p-13 exhibited an obvious increase in TLCCM. TLCCM of q-15 and p-13 were 13.41 ± 0.29 and 16.89 ± 1.05 mg/10^9^ cells, showing 31.58 and 65.65 % increase over wild-type with a statistical significant difference (*P* < 0.05), respectively. It should be noted that TLCCM per liter of q-15 was slightly, but not obviously higher than that of wild-type because of the slightly lower growth rate. In contrast, TLCCM per liter of p-13 showed an obvious increase compared with wild-type.

The increase in TLCCM of transgenic algae could either result from FAs secretion or lysis of algae which had higher intracellular lipid content. To exclude the possibility of lysis of algae, we determined protein concentration in the culture supernatant using the BCA protein assay kit. The protein concentration in culture supernatant of q-15, p-13 and cc849 was almost zero. Moreover, deformed cells and cell debris were not found in algae culture. In addition, we measured the FAs content of both supernatant and pellet corresponding to culture medium and algae, respectively, according to Scharnewski’s method [25]. The FAs contents of culture medium in q-15, p-13 and cc849 were 8.19 ± 1.02, 9.66 ± 1.42 and 2.93 ± 0.07 mg/10^9^cells, respectively, which showed 2.79 and 3.29 folds increase over the control strain. While the intracellular FAs contents of algae pellet in q-15, p-13 and cc849 were 4.8 ± 0.14, 4.9 ± 0.11 and 6.8 ± 0.22 mg/g DCW, respectively. In all, these results clearly demonstrated that FAs in culture medium was secreted not lysed from cells whose lipids were mainly TAG.

FAs secretion resulted from LACSs disruption was reported in *S. cerevisiae* when both *faa1* and *faa4* genes were simultaneously disrupted. However, independent knockdown of LACS gene (*faa1*, *faa2*, *faa3*, *faa4*, or *fat1*) showed little FAs secretion [7, 14]. We found single *cracs* knockdown was enough to induce FAs secretion in *C. reinhardtii*. So, we inferred that enhanced FAs secretion may be achieved by co-knockdown of both *cracs1* and *cracs2* in one single cell. Moreover, Scharnewski found that secreted FAs were re-imported back in stationary-phase in LACS genes disrupted yeast [25]. This is different from the phenomenon observed in our transgenic algae, as both intracellular lipid and extracellular FAs of q-15 and p-13 increased in stationary-phase cells. This difference may be because it is unnecessary for algae to re-import FAs for survival as they can synthesize carbohydrate by photosynthesis, however, yeasts have to absorb FAs when environmental nutrition was depleted in stationary-phase. This is supported by the facts that adding extra carbohydrate to stationary-phase yeast can stop FAs import and initiate export [25].

## Conclusion

In this study, two LACSs cDNAs, *cracs1* and *cracs2*, in *C. reinhardtii* were successfully cloned and characterized. Both genes have the ability of foreign FAs importation and are involved in lipid metabolic pathways, but have different substrate adaptability. Knockdown of either *cracs1* or *cracs2* in *C. reinhardtii* resulted in intracellular lipid accumulation and extracellular FAs secretion. These results confirmed the possibility of microalgae FAs secretion and may give a new strategy of low-cost oil extraction. According to our results, microalgae FAs secretion may also be achieved in other species by this method, which may promote the industrial application of microalgae biofuels in the future.

## Methods

### Strains, vectors and culture condition

*Chlamydomonas reinhardtii* cc849 (cell wall deficient strain) was obtained from the Chlamydomonas Resource Center. *S. cerevisiae* YB525 (a;*ura*3–52; *leu*2–3,112; *his*3Δ-200; *ade*2–101; *lys*2–801; *faa1*Δ::HIS3; *faa4*Δ::LYS2) was kindly provided by professor Pan Kehou, Ocean University of China. *E. coli* DH5α was used for normal DNA manipulation. Vector pJD124, a home-made vector with Hsp70-RBCS2 promoter, was used for gene expression in *C. reinhardtii*. Vector pYES2 (Invitrogen) with GAL1 promoter was used for LACS gene expression in *S. cerevisiae*.

cc849 was cultured in Tris–acetate–phosphate (TAP) medium under continuous light (60 μmol m^−2^ s^−1^) at 25 °C. Nitrogen starvation was conducted in TAP-N medium (KCl substituted for NH_4_Cl) when cell was grown to mid-logarithmic phase. To induce knockdown of *cracs*, transgenic algae p-13 and q-15 were grown to 1 × 10^5^ cells/mL in TAP and subjected to 40 °C to induce antisense *cracs* expression. Then, cells were continuously cultured at 25 °C for 48 h.

YB525 transformants were cultured in dropout uracil medium containing 0.67 % yeast nitrogen base, 0.077 % complete supplement mixture (without uracil), and 2 % dextrose or 2 % galactose at 30 °C.

### Cloning of LACSs genes and sequence analysis

The putative LACSs genes in *C. reinhardtii* were screened from NCBI genome database using nucleotide sequence from *A. thaliana*, *N. oculata* and *T. pseudonana* by BLAST. Primers listed in Additional file 1: Table S1 were designed according to BLAST results. Total RNA was isolated by Takara RNAiso Plus Kit according to its instruction. With Takara Reverse Transcriptase M-MLV, the first string of cDNA was synthesized using oligo-dT as the reversed primer according to its protocol. *Cracs1* and *cracs2* were amplified using LA Taq polymerase (Takara) with high GC buffer II by primer pairs crACSF1/crACSR1 and crACSF2/crACSR2, respectively. PCR products were then cloned into pEASY-T vector (Transgene) after purification and subjected to sequencing. Open reading frame (OFR) was predicted using ORF Finder (http://www.ncbi.nlm.nih.gov/gorf/gorf.html). Protein sequences were aligned using ClustalX and phylogenetic tree was constructed using MEGA 4.0.

### Plasmid construction and transformation

Normal molecular manipulations were operated as described by Sambrook and Russell [28]. CrACSs expression plasmids pYES2-cracs1 and pYES2-cracs2 were constructed by insertion of *cracs1* and *cracs2* into vector pYES2 under the control of galactose-inducible promoter using restriction enzyme *Kpn*I and *Xho*I. Plasmids pYES2-cracs1, pYES2-cracs2 and blank pYES2 were electroporated into yeast YB525 as described [17].

Plasmid pJD-siacs1 which was used for knockdown of *cracs1* in *C. reinhardtii* was constructed as follows. Using primer pair SiACS1F/SiACS1R, around 600 bp of *cracs1* was amplified and ligated into T vector. After digested by *Eco*RI/*Kpn*I, fragment was reversely inserted into expression vector pJD124. Plasmid pJD-siacs2 was constructed by the same method except for using primer pair SiACS2F/SiACS2R. Plasmids were introduced into *C. reinhardtii* by the glass beads agitation and transformants were selected with antibiotic zeocin [29, 30].

### Yeast complementation assay

YB525 transformants were grown to mid-log phase in dropout uracil medium containing 2 % dextrose, then harvested, and washed with 2 M sorbitol for two times. Cells were re-suspended in fresh dropout uracil medium containing 2 % galactose and then cultured for 4 h at 30 °C to induce *cracs* expression. 100 μL induced cells was then re-inoculated into dropout uracil medium containing 2 % galactose, 0.1 % Triton X-100, 25 mM cerulenin and 100 μmol/L fatty acid for 240 h. The OD_600_ of wild type and transformants were determined at last.

### Quantitative reverse transcription-PCR (qRT-PCR)

Total RNA was used to synthesize cDNA as mentioned above. Using SYBR Premix ExTaq Kits (Takara), 2 μL of cDNA was used for qRT-PCR according to its instruction on an ABI Prism 7900 Sequence Detection System. Primers used for qRT-PCR were listed in supplementary Additional file 1: Table S1 and housekeeping gene *β*-*actin* was used as the reference.

### Fluorescence microscopy

Images were captured by Laser Scanning Confocal Microscopy 710 (ZEISS, Germany).YB525 transformants were cultured as described above under induction for 16 h. 500 μL of cells were incubated with 1 μg/mL of Nile Red for 10 min with dark and then washed twice with ddH_2_O, and observed by confocal microscope with excitation at 488 nm and emission at 550 nm. Exogenetic FAs uptake was visualized using C1-BODIPY-C12 (4,4-difluoro-5-methyl-4-bora-3a,4a-diaza-s-indacene-3-dodecanoic acid) as the fatty acids analogue according to Pulsifer’s protocol [20]. The intracellular lipid accumulation in *C. reinhardtii* was observed by staining cells with BODIPY 505/515 (4,4-difluoro-1,3,5,7-tetramethyl-4-bora-3a,4a-diaza-s-indacene) as described elsewhere [31].

### Lipid analysis

As above described, algae were grown to mid-log phase, induced at 40 °C for 30 min and then normally cultured for another 48 h. Cells and culture supernatant were separately collected for lipid analysis. Collected cell pellet was dried by vacuum freezing for 36 h. 5 mg of dry cell pellet was re-suspended in 5 mL of ddH_2_O, disrupted by sonication and extracted with 1 mL of* n*-hexane for three times. Lipid extracts were dried by nitrogen blowing and dissolved in 500 μL of hexane. 45 mL of culture supernatant was extracted with 10 mL of* n*-hexane for 3 times, dried by nitrogen blowing and dissolved in 50 μL of hexane.

FAs analysis was conducted by gas chromatography/mass spectrometry (GC/MASS) according to a modified protocol described elsewhere [25]. In brief, 50 μL of lipid extracts were dried by nitrogen blowing first. After adding 800 μL of methanol, 5 μL of nonadecanoic acid (C19:0, 200 μg/mL, internal standard) and 20 μL of 1-ethyl-3-(3-dimethylaminopropylcarbodiimide) (0.1 mg/μL in methanol), the mixture was incubated at 22 °C for 2 h. Then, 400 μL of saturated NaCl was added to stop reaction. The methyl esters of FAs were extracted with 1 mL of* n*-hexane for two times, dried by nitrogen blowing, dissolved in 10 μL of CH_2_Cl_2_ and then analyzed by GC/MASS.

Total lipid analysis was performed as follows. 1 mL of NaOH–CH_3_OH solution (2 mol/L) was added to 10 mg of dry cell pellet or 50 μL of culture supernatant lipid extracts. The mixture was transferred to glass tube and incubated at 75 °C for 30 min with shaking. After cool-down, 1 mL of HCl–CH_3_OH (4 mol/L) was added and pH was adjusted to below 2.0 with HCl. Then, the reaction mixture was incubated at 75 °C for another 30 min. After that, fatty acid methyl esters were extracted with* n*-hexane, dried by nitrogen blowing, dissolved in 500 μL of CH_2_Cl_2_ and quantified by GC/MASS with C19:0 as the internal standard.

GC/MASS analysis was performed by Thermo Trace GC Ultra gas chromatograph coupled to Thermo Polaris Q mass spectrometry equipped with a HP-5MS column (30 mm × 0.25 mm, film thickness 0.25 μm) as elsewhere described [32].

## Additional files

10.1186/s13068-016-0598-7 Complete list of primers used in this study.
10.1186/s13068-016-0598-7 The detailed exon distribution of* cracs1* and* crasc2* genes in C. reinhardtii genome. (A)* cracs1* was predicted to contain 16 extons, which is in full agreement with a predicted protein cds sequence (accession numbers: XM-001702895.1) except for a G to C change in the 6th exon. (B)* cracs2 *was predicted to contain 17 extons. The 7th and 11th exons with total number of 399bp just right fell into the gap region of genomic sequence date.
10.1186/s13068-016-0598-7 Both cracs genes responded to Nitrogen starvation (NS). (A) the mRNA level of* cracs1* gradually increased with prolonged NS. (B) the mRNA level of* cracs2* decreased with prolonged NS.
10.1186/s13068-016-0598-7 Transgenic algaes and their PCR verification. (A) transgenic algaes were screened by antibiotic zeocin in TAP plate.(B) PCR verification of transformants using genomic DNA.* Lane* 1–12: transformants of* cracs1* gene knockdown.* lane* 13–24: transformants of* cracs2* gene knockdown. M: DNA marker DL2000.
10.1186/s13068-016-0598-7 Growth carves of transgenic algaes q-15, p-13 and control strain cc849. Transgenic transformants q-15(antisense knockdown of* cracs1*) and p-13(antisense knockdown of* cracs1*) presented similar growth in the early stage of culture and slightly lower growth rate in the stationary stage when they were compared with the wild type cc849.
10.1186/s13068-016-0598-7 The GenBank accession number of sequences used in Phylogenetic analysis.

## Abbreviations

LACSs: long-chain acyl-CoA synthetases
ACSs: acyl-CoA synthetases
FAs: fatty acids
TLCCM: total lipid content in culture medium
qRT-PCR: quantitative reverse transcription-PCR
GC/MASS: gas chromatography/mass spectrometry
TAP: tris–acetate–phosphate
DCW: dry cell weight
C1-BODIPY-C12: 4,4-difluoro-5-methyl-4-bora-3a,4a-diaza-s-indacene-3-dodecanoic acid
BODIPY 505/515: 4,4-difluoro-1,3,5,7-tetramethyl-4-bora-3a,4a-diaza-s-indacene
