# Supplementary material for: Characterization of long-chain acyl-CoA synthetases which stimulate secretion of fatty acids in green algae Chlamydomonas reinhardtii
Source: Biotechnol Biofuels. 2016 Aug 31;9(1):184. doi: 10.1186/s13068-016-0598-7 (PMC5007677; doi:10.1186/s13068-016-0598-7)
Supplement: Supplementary file 1 — 10.1186/s13068-016-0598-7 Complete list of primers used in this study. [file 13068_2016_598_MOESM1_ESM.docx]

**Table S1.**

| **Primer name** | **PRIMER SEQUENCE(5’-3’)** | **description** |
| --- | --- | --- |
| crACSF1 | GGGGTACCATGGAGCCGGCCCGCCCGGCTGGT | Primers used for cloning of *cracs1* |
| crACSR1 | CCGCTCGAGTCACGACGCCGGCGCTC |  |
| crACSF2 | GGGGTACCATGGCCCCGACAGCGGGG | Primers used for cloning of *cracs2* |
| crACSR2 | CCGCTCGAGTCAGTGTGTGGCGGGCATTGCGGCGT |  |
| ActinF | ACCCCGTGCTGACTG | qRT-PCR primer of reference gene β-actin |
| ActinR | ACGTTGAAGGTCTCGAACG |  |
| QACS1F | CGTCGGATACTGGCAGGGTG | qRT-PCR primer of *cracs1* |
| QACS1R | ATAAGCCGGGACAGGAAGC |  |
| QACS2F | CCAAGTTTCCGCACATTCCG | qRT-PCR primer of *cracs2* |
| QACS2R | CAGCCACTCCTTGCAGTTTGGACG |  |
| SiACS1F | CGGAATTCGTCGGCGTCCGCGGCGGCGCC | primer used for construction of antisense plasmid pYES2-cracs1 |
| SiACS1R | GGGGTACCATGGAGCCGGCCCGCCCGGCTGGT |  |
| SiACS2F | CGGAATTCCACCGTCAGCATGGTGGGCAGCA | primer used for construction of antisense plasmid pYES2-cracs2 |
| SiACS2R | GGGGTACCATGGCCCCGACAGCGGGG |  |
| QSiACS1F | CGTCGGATACTGGCAGGGTG | qRT-PCR primer of *cracs1* expression in transgenetic algae |
| QSiACS1R | AAGATAAGCCGGGACAGGAAGC |  |
| QSiACS2F | GGCAACCGCCACCTGTCGTA | qRT-PCR primer of *cracs2* expression in transgenetic algae |
| QSiACS2R | CGAGGCACCGAGGCGAAGAT |  |

underlined bases indicate the restriction enzyme sites
