# Supplementary figures and images for: Characterization of long-chain acyl-CoA synthetases which stimulate secretion of fatty acids in green algae Chlamydomonas reinhardtii
Source: Biotechnol Biofuels. 2016 Aug 31;9(1):184. doi: 10.1186/s13068-016-0598-7 (PMC5007677; doi:10.1186/s13068-016-0598-7)

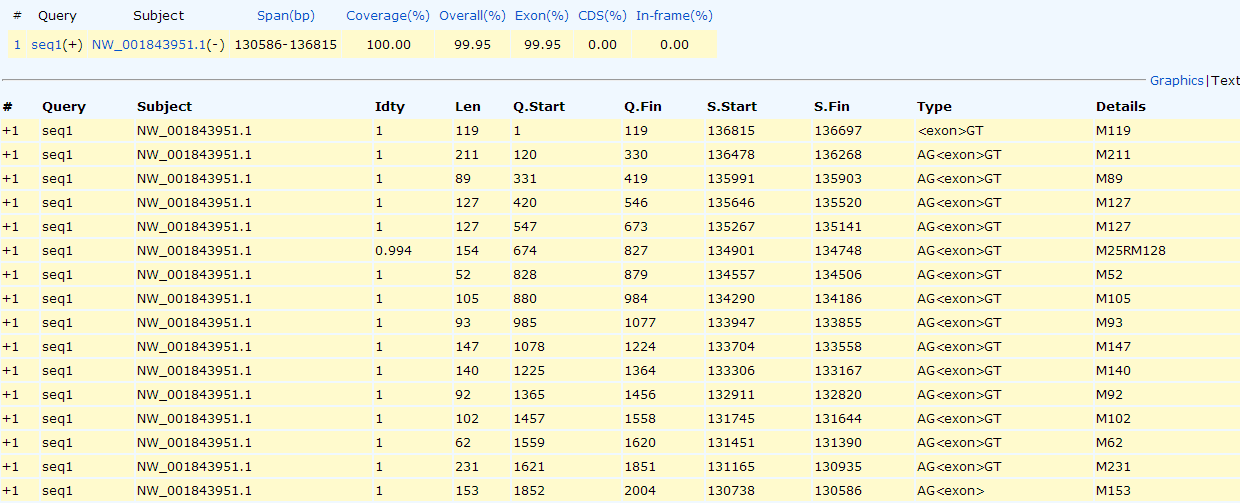


**A**
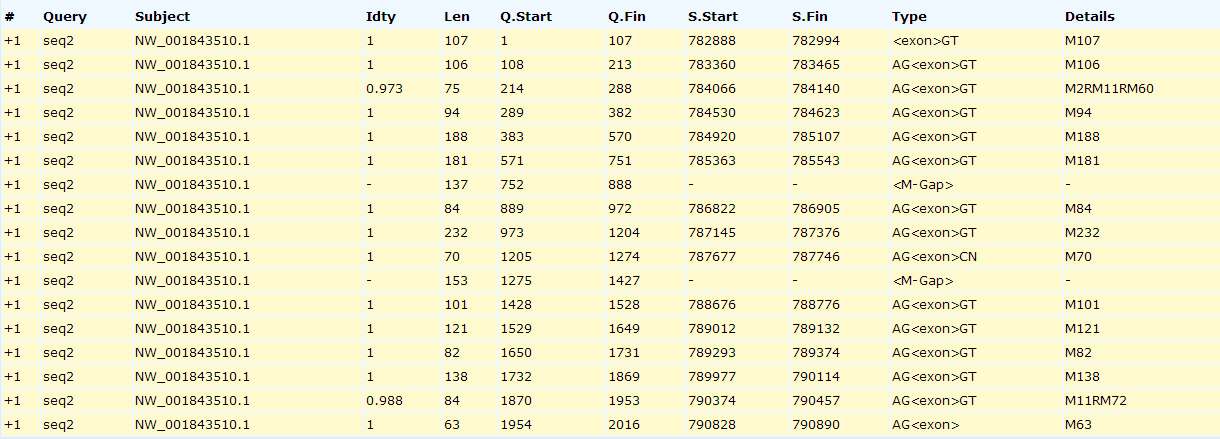


**B**

**Fig.S1**

Supplement: Supplementary file 2 — 10.1186/s13068-016-0598-7 The detailed exon distribution of cracs1 and crasc2 genes in C. reinhardtii genome. (A) cracs1 was predicted to contain 16 extons, which is in full agreement with a predicted protein cds sequence (accession numbers: XM-001702895.1) except for a G to C change in the 6th exon. (B) cracs2 was predicted to contain 17 extons. The 7th and 11th exons with total number of 399bp just right fell into the gap region of genomic sequence date. [file 13068_2016_598_MOESM2_ESM.docx]

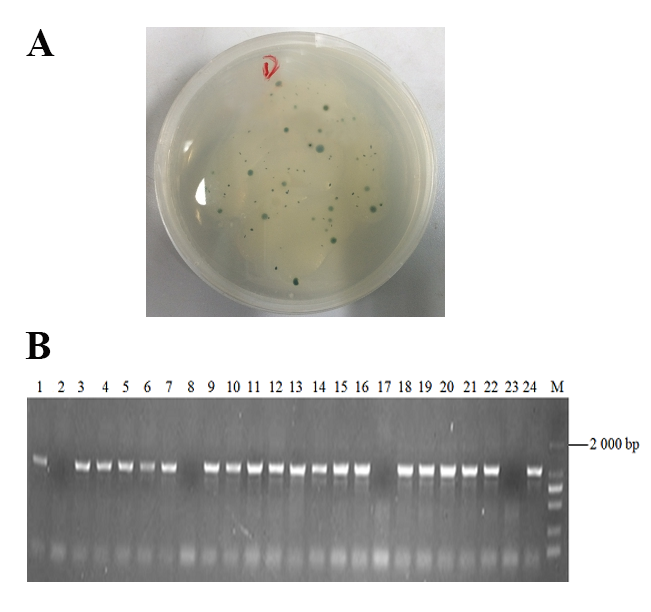
**Fig. S3**

Supplement: Supplementary file 4 — 10.1186/s13068-016-0598-7 Transgenic algaes and their PCR verification. (A) transgenic algaes were screened by antibiotic zeocin in TAP plate.(B) PCR verification of transformants using genomic DNA. Lane 1–12: transformants of cracs1 gene knockdown. lane 13–24: transformants of cracs2 gene knockdown. M: DNA marker DL2000. [file 13068_2016_598_MOESM4_ESM.docx]

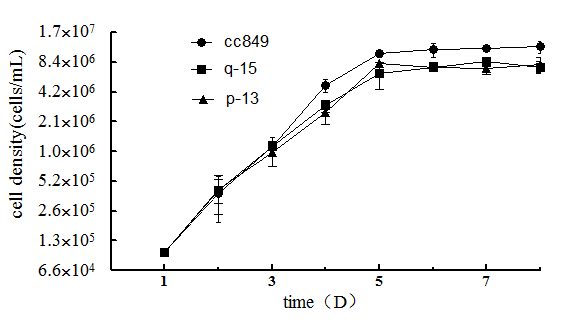


**Fig. S4**

Supplement: Supplementary file 5 — 10.1186/s13068-016-0598-7 Growth carves of transgenic algaes q-15, p-13 and control strain cc849. Transgenic transformants q-15(antisense knockdown of cracs1) and p-13(antisense knockdown of cracs1) presented similar growth in the early stage of culture and slightly lower growth rate in the stationary stage when they were compared with the wild type cc849. [file 13068_2016_598_MOESM5_ESM.docx]
