# Supplementary material for: Characterization of long-chain acyl-CoA synthetases which stimulate secretion of fatty acids in green algae Chlamydomonas reinhardtii
Source: Biotechnol Biofuels. 2016 Aug 31;9(1):184. doi: 10.1186/s13068-016-0598-7 (PMC5007677; doi:10.1186/s13068-016-0598-7)
Supplement: Supplementary file 3 — 10.1186/s13068-016-0598-7 Both cracs genes responded to Nitrogen starvation (NS). (A) the mRNA level of cracs1 gradually increased with prolonged NS. (B) the mRNA level of cracs2 decreased with prolonged NS. [file 13068_2016_598_MOESM3_ESM.docx]

A B

**Fig. S2**
