# Supplementary material for: Characterization of long-chain acyl-CoA synthetases which stimulate secretion of fatty acids in green algae Chlamydomonas reinhardtii
Source: Biotechnol Biofuels. 2016 Aug 31;9(1):184. doi: 10.1186/s13068-016-0598-7 (PMC5007677; doi:10.1186/s13068-016-0598-7)
Supplement: Supplementary file 6 — 10.1186/s13068-016-0598-7 The GenBank accession number of sequences used in Phylogenetic analysis. [file 13068_2016_598_MOESM6_ESM.docx]

Table S2

| Species | Name | Accession NO. |
| --- | --- | --- |
| *Arabidopsis thaliana* | AtLACS1 | AAM28868.1 |
| *Arabidopsis thaliana* | AtLACS2 | AAM28869.1 |
| *Arabidopsis thaliana* | AtLACS3 | AAM28870.1 |
| *Arabidopsis thaliana* | AtLACS4 | AAM28871.1 |
| *Arabidopsis thaliana* | AtLACS5 | AAM28872.1 |
| *Arabidopsis thaliana* | AtLACS6 | AAM28873.1 |
| *Arabidopsis thaliana* | AtLACS7 | AAM28874.1 |
| *Arabidopsis thaliana* | AtLACS8 | AAM28875.1 |
| *Arabidopsis thaliana* | AtLACS9 | AAM28876.1 |
| *Saccharomyces cerevisiae* | ScFAA1 | NP_014962.3 |
| *Saccharomyces cerevisiae* | ScFAA4 | NP_013974.1 |
| *Saccharomyces cerevisiae* | ScVLACS | AAC17118.1 |
| *Homo sapiens* | HsVLACS | NP_003636.2 |
| *Homo sapiens* | HsLACS3 | NP_004448.2 |
| *Brassica napus* | BnACS | CAA64327.1 |
| *Rattus norvegicus* | RnACS | BAA22195.1 |
| *Rattus norvegicus* | RnACS5 | BAA33581.1 |
| *Phaeodactylum tricornutum* | PtLACS1 | AGZ03882.1 |
| *Phaeodactylum tricornutum* | PtLACS3 | AGZ03884.1 |
| *Phaeodactylum tricornutum* | PtLACS5 | AGZ03886.1 |
| *Thalassiosira pseudonana* | TpLACS | XP_002293417.1 |
| *Brassica napus* | BnACS | CAA64327.1 |
| *Pseudomonas aeruginosa* | PaLACS | NP_251989.1 |
